# Correlation of Preoperative MRI and Shoulder-Specific Tests With Intraoperative Findings in Rotator Cuff Tears

**DOI:** 10.7759/cureus.80951

**Published:** 2025-03-21

**Authors:** Emre Arikan, Yasin E Kaya, Metin Celik, Mahmut Kurtbogan, Ilhan Celik, Tacettin Ayanoglu

**Affiliations:** 1 Department of Orthopaedics and Traumatology, Bursa Ali Osman Sonmez Oncology Hospital, Bursa, TUR; 2 Department of Orthopaedics and Traumatology, Bolu Abant Izzet Baysal University, Bolu, TUR; 3 Department of Orthopaedics and Traumatology, Malatya Training and Research Hospital, Malatya, TUR; 4 Department of Orthopaedics and Traumatology, Alanya Alaaddin Keykubat University, Antalya, TUR

**Keywords:** arthroscopy, magnetic resonance imaging, physical examination, rotator cuff tears, shoulder pain

## Abstract

The aim of this study was to compare preoperative magnetic resonance imaging (MRI) findings, shoulder tests, and intraoperative observations in patients who underwent arthroscopic rotator cuff (RC) repair at a single center in Turkey. A total of 162 patients diagnosed with rotator cuff tears (RCTs) were included in this study. The physical examination involved the Jobe, Neer, and drop-arm tests. For patients undergoing shoulder arthroscopy, tear type and stage were evaluated intraoperatively and classified as partial-thickness or full-thickness tears. Preoperative assessments using MRI documented tear types and stages. Test results and imaging findings were compared to intraoperative observations to ensure diagnostic accuracy. A study of shoulder arthroscopy patients (129 right, 33 left; mean age 58.19 ± 9.20 years) found that 77.2% had full-thickness tears and 22.8% had partial tears on preoperative MRI. Significant differences were noted in the drop-arm test results (p = 0.003). MRI proved less reliable in localizing partial RCTs. Grade changes in full-thickness tears (Patte classification, p < 0.001) and partial tears (Ellman classification, p = 0.018) were statistically significant between preoperative and intraoperative evaluations. Certain specialized shoulder tests demonstrate strong diagnostic accuracy for specific conditions; however, their effectiveness may be limited when applied in isolation. MRI is a reliable tool for diagnosing RCTs; however, its sensitivity is reduced when detecting partial tears. This discrepancy suggests that both full-thickness and partial tears may exhibit more advanced pathology at the time of surgical intervention than initially indicated by preoperative imaging.

## Introduction

Rotator cuff tears (RCTs) are one of the most common causes of shoulder pain, diagnosed through a combination of patient history, clinical examination, and diagnostic imaging, while shoulder pain ranks as the third most common cause of musculoskeletal discomfort [1,2]. RCTs are a frequent shoulder injury that can severely limit joint movement and impact the daily lives of affected individuals. Some RCTs present with symptoms, while others remain asymptomatic [3,4].

Arthroscopy is now regarded as the "gold standard" for diagnosing shoulder pathologies [5]. RCTs, commonly seen in individuals over 50, cause shoulder pain, weakness, and functional limitations, often requiring surgical intervention when symptoms progress or conservative treatments fail [6]. Arthroscopic repair, the most widely used method, allows for minimal tissue damage and clear visualization of joint structures through small incisions, enabling precise tendon reattachment. For larger tears, open surgery or double-row techniques may be employed to enhance tissue stability and improve outcomes. Proper technique and patient selection are crucial for successful rotator cuff (RC) repair, ultimately restoring shoulder function and relieving pain [6].

Magnetic resonance imaging (MRI), with its superior soft tissue resolution and multi-planar imaging capabilities, is the preferred method for preoperative evaluation of RC conditions [7,8]. MRI provides crucial information on tendon retraction, muscle atrophy, fatty degeneration, and coracoacromial impingement, which can have significant prognostic value [9].

Physical examination remains essential for accurate diagnosis and treatment planning. Specific physical examination techniques help determine the location and extent of RCTs.

The aim of this study, conducted with 162 patients, was to compare MRI findings with intraoperative observations in patients who underwent arthroscopic RC repair and to retrospectively evaluate the diagnostic accuracy of RC examination tests.

## Materials and methods

Ethics

This retrospective study received approval from the Abant İzzet Baysal University (AIBU) TF Ethics Committee (approval date: August 16, 2019; decision no: 2019/161). Data were collected from 162 patients who underwent surgery for RCTs between 2015 and 2019 at the Clinical Sciences Laboratory, Faculty of Medicine, AIBU, Bolu, Turkey. Written informed consent was obtained from all patients after explaining the nature, purpose, and expected outcomes of the study.

Study design

A total of 162 patients who underwent surgery for RCTs between 2015 and 2019 were included in this study. The study criteria encompassed patients who experienced persistent symptoms despite at least six months of conservative treatment and who had no additional shoulder pathologies aside from the RC lesion.

Exclusion criteria

Exclusion criteria included cases with concomitant adhesive capsulitis, cervical disc pathology, calcific tendinitis, snapping scapula, additional intra-articular pathologies (such as RC tears accompanied by Bankart or superior labrum from anterior to posterior tear (SLAP) lesions), suprascapular neuropathy, scapular dyskinesia, deltoid atony, and inflammatory joint diseases.

Preoperative assessment

**Figure 1:**
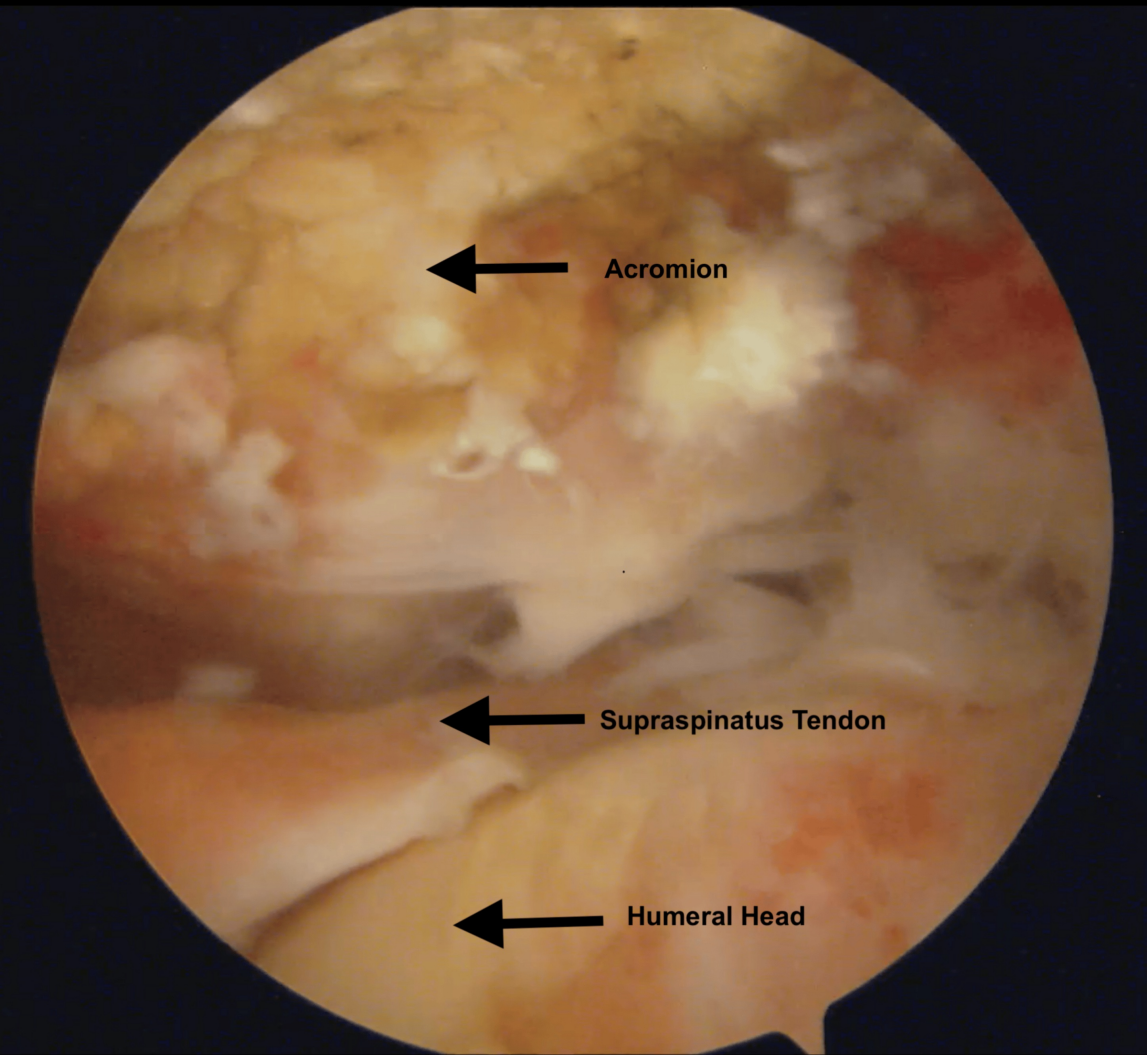
Patte classification stage 3 intraoperative rotator cuff rupture

Both preoperative MRI and physical examination findings were documented, along with intraoperative images from posterior portal views (Figure 1). Physical examination assessments, including the drop-arm test, Jobe test, and Neer Ttst, were conducted by an experienced orthopedic surgeon, and video recordings of these evaluations were made. These tests were performed according to standard examination protocols [2,10]. Positive test results supported a preliminary diagnosis of RCT.

MRI protocol

MRI was performed preoperatively using a 1.5 T Siemens Magnetom Symphony (Siemens Healthineers, Erlangen, Germany) scanner with dedicated 16-channel system coils in standard positioning, studying different sequences including T1W, T2W, T2 STIR, and PDW. MRI scans of the entire shoulder were conducted to evaluate findings related to RC pathology for each shoulder. All MRIs were evaluated by a single radiologist who was unaware of the clinical findings.

Intraoperative evaluation

During surgery, all patients underwent arthroscopic evaluation of the glenohumeral joint and subacromial space. Tear size, tear type, and tear location were assessed through posterior portal imaging by an experienced shoulder surgeon, with findings documented and recorded on video.

Clinical physical examination and MRI results were then compared with intraoperative arthroscopic findings.

Statistical analysis

​​For numerical variables, descriptive statistics are presented as either the mean and standard deviation (SD) or median with minimum and maximum values, depending on the distribution. For categorical variables, frequencies and percentages are reported. The normality of the data was assessed using the Kolmogorov-Smirnov test. For comparisons between two groups, the independent samples t-test was used when the assumptions of normality and homogeneity of variance were met. If the assumptions were violated, the Mann-Whitney U test was applied. For categorical variables, the Chi-square test was used to evaluate significant relationships between groups. The McNemar-Bowker test was applied to assess changes over time. Cohen’s kappa coefficient was calculated to evaluate agreement and correlation between the two assessment methods. Statistical significance was defined as a p-value less than 0.05. All analyses were performed using IBM SPSS Statistics for Windows, Version 21.0 (released 2012, IBM Corp., Armonk, NY).

## Results

Demographic, clinical, and preoperative findings

In a cohort of 162 patients who underwent shoulder arthroscopy, 112 were female and 50 were male. The right shoulder was affected in 129 patients, while the left shoulder was affected in 33 patients(Table 1). The mean age of the patients was 58.19 ± 9.20 years, ranging from 30 to 76 years. Preoperative MRI findings revealed that 77.2% (125 patients) had full-thickness RCTs, while 22.8% (37 patients) had partial-thickness RCTs. A statistically significant difference was observed in the drop-arm test results between these groups (p = 0.003) (Table 2). Specifically, 64.8% of the patients with full-thickness tears exhibited positive test results, while a majority (62.2%) of patients with partial tears had negative results. However, no significant difference was found between the groups in the outcomes of the Jobe and Neer tests.

**Table 1:** Demographic data ± represents the standard deviation (SD). RCT: rotator cuff tear

Total no. of patients	Female	Male	Right shoulder affected	Left shoulder affected	Mean age	Age range	Full-thickness RCTs	Partial-thickness RCTs
162	112 (69.1%)	50 (30.9%)	129 (79.6%)	33 (20.4%)	58.19 ± 9.20 years	30 - 76 years	125 (77.2%)	37 (22.8%)

**Table 2:** Shoulder-specific tests comparison results by groups The p-value was determined using the chi-square test. Statistical significance was defined as a p-value less than 0.05. The Jobe test (assesses rotator cuff tendon pathology), Neer test (evaluates subacromial impingement), and drop-arm test (detects rotator cuff tears).

Specific shoulder examination test	Specific shoulder test result	Full-thickness tears	Partial-thickness tears	p
Jobe test	Negative	18 (14.4)	8 (21.6)	0.293
	Positive	107 (85.6)	29 (78.4)	
Neer test	Negative	18 (14.4)	7 (18.9)	0.504
	Positive	107 (85.6)	30 (81.1)	
Drop-arm test	Negative	44 (35.2)	23 (62.2)	0.003
	Positive	81 (64.8)	14 (37.8)	

Preoperative versus intraoperative findings and variability

**Figure 2:**
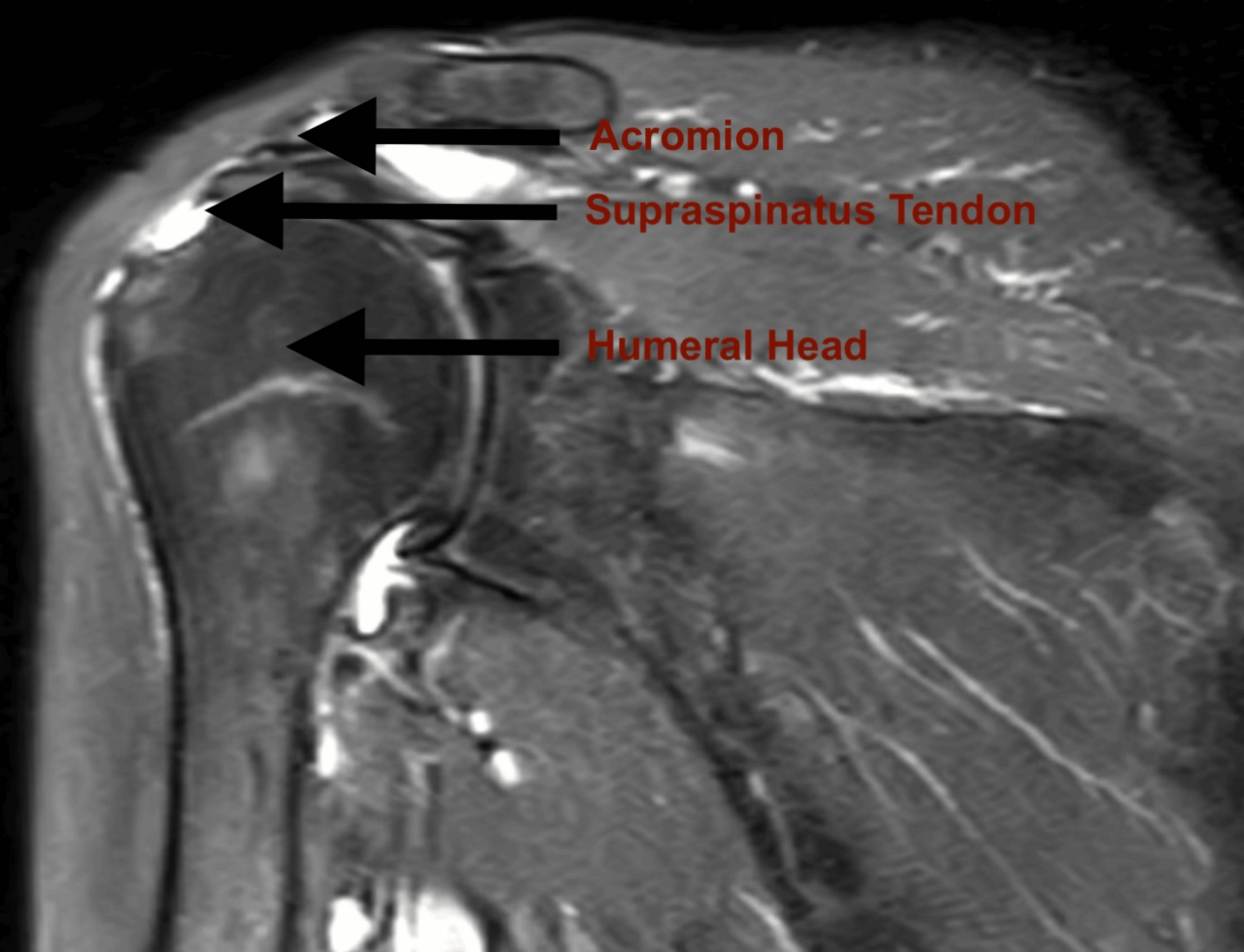
Patte classification stage 2 shoulder magnetic resonance imaging

Preoperative MRI identified 77.2% of patients as having full-thickness RCTs, while intraoperative evaluation confirmed full-thickness tears in 84.6% of cases (Table 3). For partial-thickness RCTs, MRI showed 22.8% of patients, but intraoperative findings identified 15.4% of cases (Table 2). Among partial tears, MRI classified 70.3% as articular-side tears and 29.7% as bursal-side tears, whereas intraoperative evaluation revealed 80% as articular-side and 20% as bursal-side (Table 4). A comparison of full-thickness RCTs identified by preoperative MRI with intraoperative findings in the same patient demonstrated notable variability in the classification of these tears (Tables 5) (Figures 1, 2).

**Table 3:** Comparison of preoperative and intraoperative rotator cuff pathologies (N = 162)

Tear type	Preoperative		Intraoperative	
	Number	Percent	Number	Percent
Full-thickness tears	125	77.2	137	84.6
Partial-thickness tears	37	22.8	25	15.4

**Table 4:** Regional distribution of rotator cuff partial-thickness tears

Tear side	Preoperative (N = 37)		Intraoperative (N = 25)	
	Number	Percent	Number	Percent
Articuler-sided rotator cuff tear	26	70.3	20	80.0
Bursal-sided rotator cuff tear	11	29.7	5	20.0

**Table 5:** Descriptive statistics for Patte classification findings

Patte classification grade		Preoperative		Intraoperative	
		Number	Percent	Number	Percent
Full-thickness tear	1	43	34.4	30	24.0
	2	49	39.2	27	21.6
	3	33	26.4	68	54.4
	Total	125	100.0	125	100.0
Partial-thickness tear	1	0	0.0	7	77.8
	2	0	0.0	1	11.1
	3	0	0.0	1	11.1
	Total	0	0.0	9	100.0

Grading changes in RCTs

In full-thickness RCTs, grading changes from preoperative to intraoperative assessment were statistically significant according to the Patte classification (p < 0.001) (Table 6). Among patients initially graded as stage 1, 20% progressed to stage 2, and 9.3% advanced to stage 3 intraoperatively. Similarly, of those classified as stage 2 preoperatively, 63.3% were identified as stage 3 during surgery. The overall concordance rate between preoperative and intraoperative findings using the Patte classification was 48.9% (Table 6).

**Table 6:** Changes in Patte classification of full-thickness tears over time The p-value was determined using the chi-square test. The values in parentheses represent percentage distributions.

Intraoperative	Preoperative						
	Grade	1	2	3	Total	P	Kappa
	1	30 (69.8 )	0	0	30 (24.0)	<0.001	0.489
	2	9 (20.9)	18 (36.7)	0	27 (21.6)	-	-
	3	4 (9.3)	31 (63.3)	33 (100.0)	68 (54.4)	-	-
	Total	43 (34.4)	49 (39.2)	33 (26.4)	125	-	-

For partial-thickness RCTs, significant grading changes were observed from preoperative to intraoperative evaluations according to the Ellman classification (p = 0.018). Specifically, one of the two patients initially graded as stage 1 was reclassified as stage 2 during surgery, while 72.7% of patients initially graded as stage 2 progressed to stage 3 intraoperatively (Table 7). The concordance rate between preoperative and intraoperative evaluations for the Ellman classification was κ = 0.282 (Table 8).

**Table 7:** Descriptive statistics for ellman classification findings

Changes in findings		Preoperative		Intraoperative	
Ellman classification	Grade	Number	Percent	Number	Percent
	1	2	5.4	2	8.0
	2	12	32.4	3	12.0
	3	23	62.2	20	80.0
	Total	37	100.0	25	100.0

**Table 8:** Change in Ellman classification of partial-thickness tears over time The p-value was determined using the chi-square test. The values in parentheses represent percentage distributions.

Intraoperative	Preoperative						
	Grade	1	2	3	Total	p	Kappa
	1	1 (50.0)	1 (9.1)	0	2 (8.0)	0,018	0,282
	2	1 (50.0)	2 (18.2)	0	3 (12.0)	-	-
	3	0 (0.0)	8 (72.7)	12 (100.0)	20 (80.0)	-	-
	Total	2 (8.0)	11 (44.0)	12 (48.0)	25	-	-

## Discussion

The aim of this study is to compare preoperative MRI findings and shoulder tests with intraoperative findings in patients undergoing shoulder arthroscopy. The cohort consisted of 162 patients, with 77.2% diagnosed with full-thickness RCTs and 22.8% with partial-thickness RCTs. Significant differences were observed in the results of the drop arm test between these two groups (p = 0.003). In addition, comparisons between preoperative and intraoperative classifications revealed notable variability, particularly in the grading of full-thickness RCTs, with a concordance rate of 48.9% for the Patte classification. These findings highlight the importance of intraoperative evaluation in accurately assessing RCT severity.

The shoulder joint is one of the most mobile joints in the body, making it susceptible to trauma. The third most common cause of pain in the musculoskeletal system is shoulder pain [11]. Among the diseases that cause shoulder pain, the most common cause is diseases caused by RC pathologies [12]. The incidence of RCTs increases with age [12]. The increase in shoulder pathologies with aging develops due to tendon degeneration and progressive tendon damage [13,14]. In our study, the mean age of the patients was determined as 58.19 ± 9.20 (30-76 age). These findings support the increase in shoulder pain with age, consistent with the literature [13,15].

In a study of 334 people, the sensitivity for partial RC tears in MRI was 51.6%, the specificity was 77.2%, the sensitivity for the Jobe test was 64.1%, and the specificity was 43.2%. The sensitivity for the Neer test was 76.7%, and the specificity was 46.6%. When MRI data, Jobe test, and Neer test were evaluated together, the sensitivity was 46.9% and the specificity was 85.4%. According to this study, the diagnostic accuracy of MRI and clinical tests (Jobe test and Neer test) alone is limited to detect partial RCTs [16]. In conclusion, a negative test does not exclude RC and/or subacromial impingement syndrome, but we can say that a positive test indicates the presence of shoulder pathologies despite having a wide etiology range. Isolated shoulder-specific tests are not sufficient to establish an accurate diagnosis. Combining these special tests can enhance the diagnostic accuracy of a shoulder physical examination. If needed, patient history and diagnostic imaging should also be taken into account to improve accuracy [10]. The Jobe test demonstrated the highest combined sensitivity, whereas the drop-arm sign exhibited the highest combined specificity [17]. Since no single clinical test is reliable enough to diagnose posterosuperior RCTs on its own, healthcare providers should take into account a combination of patient factors, clinical assessments, and imaging techniques to establish a diagnosis and determine the most suitable treatment plan [17]. The most common lesions in patients presenting with shoulder pain are RC lesions and supraspinatus lesions among RC lesions [18]. Jobe test, which is one of the tests detecting supraspinatus lesions, was found to be positive at a higher rate than other tests in all groups. However, the drop-arm test was found to be lower. As known from previous studies, the drop-arm test is a more sensitive examination method, especially in relation to the size of the rupture [14]. In our study, the Jobe test was positive in 84% of all groups, and the most common supraspinatus lesions were identified, consistent with the literature. When partial- and full-thickness RCTs are evaluated separately, there is a statistically significant difference between the groups according to the results of the drop arm test (p = 0.003). While 64.8% of the complete tears are “positive," the majority of the partial tears are “negative” with 62.2%. According to the Jobe and Neer test results, no significant difference was found between the partial- and full-thickness tear groups. 

Preoperative MRI demonstrated a high level of agreement with intraoperative findings in assessing tear type and location; however, the greatest discrepancies were observed in high-grade partial-thickness tears [19]. Contrary to previous literature, MRI frequently overestimated tear size, with only a weak correlation to intraoperative measurements [19].

Evaluating the MRI and arthroscopic correlation of geometric patterns for RCTs as described by Burkhart, Sela et al. found a significant k value of 0.682 for the concordance between arthroscopy and MRI findings in his study [20]. In our study, for full-thickness RCTs, the k-value was found to be 0.489 for the agreement between arthroscopic intraop images and preop MRI images, and it was evaluated as "moderately compatible." For partial RCTs, the k value for the agreement between arthroscopic intraoperative images and preoperative MRI images was found to be 0.282, and it was evaluated as "low-intermediate level compatible". The low to moderate agreement between preoperative findings and intraoperative findings in patients with partial-thickness RCTs indicates that orthopedic surgeons need to be more cautious in predicting this condition.

In a study of 82 patients by Sharma et al., MRI showed high specificity and sensitivity for RC pathologies, 93.1% for full-thickness tears and 91.1% for partial-thickness tears [21]. It correlates with arthroscopic findings. Arthroscopic findings are the gold standard in RC pathologies [21].

In our study, a full-thickness RC tear is seen in 77.2% of the patients in preoperative MRI, while a full-thickness RC tear is seen in 84.6% of the patients in the intraoperative image. While partial RC tear is seen in 22.8% of the patients, 15.4% of partial RCTs are seen in intraoperative imaging.

In preoperative imaging of partial RCTs, 70.3% of the patients are on the articular side, 29.7% on the bursal side, and on intraoperative imaging, 80% of the patients are on the articular side and 20% are on the bursal side. In our study, articular side tears are more common, consistent with the literature. With conservative treatment in full-thickness RCTs, shoulder pain and shoulder dysfunction are frequently observed. It progresses in 18-24% of patients within two years in full-thickness tears that are not treated and followed conservatively [22]. Progression factors for untreated tears are age over 60, full-thickness tears, and fatty muscle degeneration of the RC muscles [23]. A study involving 122 patients suggests that full-thickness RCTs managed conservatively should be monitored more closely, as they tend to progress more rapidly than partial-thickness tears [24].

Clinical examination demonstrates 100% sensitivity and 73.8% specificity in detecting RCTs, although its diagnostic accuracy can vary depending on examiner bias, limiting its diagnostic scope [25]. MRI, on the other hand, shows 92.85% sensitivity and 98.8% specificity in detecting RCTs, with shoulder MRI exhibiting higher agreement with arthroscopy than clinical examination for the appearance of the subscapularis, supraspinatus, infraspinatus, teres, and biceps tendons [25]. The results of MRI in identifying RC pathologies are comparable to those of arthroscopy [25].

It has been shown that repairable tears can transform into irreparable tears within four years after conservative follow-up. Eran et al. showed improvement in the size of the tear in 10% of patients with symptomatic partial tears and in 50% of patients with full-thickness tears after two years of follow-up [23]. Denis et al. found long-term RCT arthropathy in untreated RCTs [26]. Ori et al. showed that half of small full-thickness RCTs under 60 years of age, which were followed conservatively, grew within three years [27]. However, the relationship between the RCTs and the development of symptoms is not fully understood.

Preoperative MRI and intraoperative images of full-thickness RCTs, in the evaluation of Patte classification in the frontal plane, it is seen that some of the tears in grades 1 and 2 preoperatively turn into grade 3 and the tears progress. Grade changes according to the Patte classification before and at the time of the operation in those with full-thickness tears were statistically significant (p < 0.001). Of the people who were grade 1 before the operation, 20% became grade 2 and 9.3% grade 3. Likewise, 63.3% of people who were grade 2 before the operation became grade 3. The Patte classification agreement before and during the operation was 48.9%. According to the Kappa value, there is moderate agreement. Preoperative MRI and intraoperative images of partial RCTs, in the evaluation of the Ellman classification in the frontal plane, show that some of the tears in grades 1 and 2 preoperatively turn into grade 3, and the tears progress. While 12 patients were evaluated as partial RCTs on preoperative MRI images, they were detected as full-thickness RCTs at various stages in intraoperative images. Grade changes according to the Ellman classification before and during the operation in those with partial-thickness tears were statistically significant (p = 0.018). One of the two people who were in grade 1 before the operation became grade 2. Likewise, 72.7% of people who were grade 2 before the operation became grade 3. Ellman classification agreement before and during the operation was 28.2%. These findings suggest that there are significant differences between preoperative MRI and intraoperative evaluation and that some RCTs may be more advanced than expected at the time of surgery. Considering that RCTs may progress and present at more advanced stages during surgery, surgeons should take this variability into account when formulating their surgical strategies.

In a study by Eren et al., the findings highlighting the impact of debridement on tear size and pattern changes indicate that larger tears may be encountered intraoperatively compared to preoperative MRI, and there is an inconsistency between surgeons and radiologists in tear pattern assessment; therefore, these differences should be considered in preoperative planning [28]. Radiological measurements in our study were made by a single radiologist unaware of the clinical findings.

Examining the seven parameters of shoulder MRI in a study involving 131 patients may highlight the challenges of achieving a complete repair of an RCT. This can be particularly relevant for less experienced orthopedic surgeons and could assist in evaluating alternative surgical approaches [29]. In our study, we aimed to identify predictive factors to minimize the risk of inadequate repair. We aimed to contribute to orthopedic arthroscopic surgery and the existing literature.

Limitations

However, our study has certain limitations. One of the primary limitations is the relatively small sample size, which may affect the generalizability of the findings. In addition, the study was conducted in a single-center setting, potentially limiting the external validity of the results. Furthermore, the retrospective nature of the study inherently introduces certain biases, such as selection bias and incomplete data retrieval. Nevertheless, a key strength of this research lies in its comparative design, as it specifically includes symptomatic patients with shoulder pain, allowing for a more clinically relevant analysis. Moreover, the study was meticulously conducted using a well-documented archival system, ensuring the reliability of the collected data. Despite these limitations, the findings are expected to make a meaningful contribution to the existing literature by providing novel insights into the relationship under investigation.

## Conclusions

Certain specialized shoulder tests (drop-arm test) demonstrate strong diagnostic accuracy for specific conditions; however, their effectiveness may be limited when applied in isolation. MRI is a highly effective modality for detecting shoulder lesions, with notably higher diagnostic accuracy for full-thickness RCTs compared to partial-thickness tears. This discrepancy suggests that both full-thickness and partial tears may exhibit more advanced pathology at the time of surgical intervention than initially indicated by preoperative imaging.
